# Honoring the Good Parent Intentions of Courageous Parents: A Thematic Summary from a US-Based National Survey

**DOI:** 10.3390/children7120265

**Published:** 2020-12-01

**Authors:** Meaghann S. Weaver, Marie L. Neumann, Blyth Lord, Lori Wiener, Junghyae Lee, Pamela S. Hinds

**Affiliations:** 1 Department of Pediatrics, Division of Pediatric Palliative Care, Children’s Hospital and Medical Center, 8200 Dodge Street, Omaha, NE 68114, USA; marie.neumann@unmc.edu; 2 Courageous Parents Network, Newton, MA 02458, USA; blord@courageousparentsnetwork.org; 3 Pediatric Oncology Branch, Center for Cancer Research, National Cancer Institute (NCI), National Institutes of Health (NIH), Bethesda, MD 20892, USA; wienerl@mail.nih.gov; 4 Department of Biostatistics, University of Nebraska Medical Center, Omaha, NE 68198, USA; Junghyae.lee@unmc.edu; 5 Department of Nursing Science, Professional Practice & Quality, Children’s National Health System, Washington, DC 20010, USA; PSHinds@childrensnational.org; 6 Department of Pediatrics, The George Washington University, Washington, DC 20010, USA

**Keywords:** pediatric, pediatric palliative care, complex medical needs, communication, parenting, family

## Abstract

Background: Parents of children with complex medical needs describe an internal, personal definition of “trying to be a good parent” for their loved child. Gaps exist in the current “good parent concept” literature: (1) When the idea of “trying to be a good parent” comes into existence for parents, (2) How parents’ definition of “being a good parent” may change over time and may influence interactions with the child, and (3) Whether parents perceive attainment of their personal definition. Aim: The purpose of this study was to explore these current gaps in the “good parent concept” knowledge base from the perspective of parents of children with chronic or complex illness. Materials and Methods: These themes were explored through a 63-item, mixed-method web-based survey distributed by the Courageous Parents Network (CPN), an organization and online platform that orients, educates, and empowers families and providers caring for seriously ill children. Results: The term “trying to be a good parent” resonated with 85% of the 67 responding parents. For the majority of parents, the concept of “being a good parent” started to exist in parental awareness before the child’s birth (70.2%) and evolved over time (67.5%) to include less judgment and more self-compassion. Parents identified their awareness of their child’s prognosis and changing health as influential on their “trying to be a good parent” concept. Parental advocacy, child’s age, and duration of illness were reported as influencing parental perceptions of having achieved their definition of “being a good parent”. Conclusions: Familiarity with parental perspectives on their parenting goodness and goals is a necessary core of family-centric health care.

## 1. Introduction

Parents hold an internal definition of “being a good parent” for a child with a medical illness, and this unique definition influences parental identity, decision-making, and relational congruence with the child [1,2]. In a qualitative study of parents with children with terminal cancer, parents described themselves as “trying to be a good parent in making care decisions in the child’s best interest” [3]. Since publication of the original “good parent concept” qualitative paper over a decade ago, care teams have partnered with parents of pediatric patients across settings to further explore the meaning and impact of this guiding “trying to be a good parent” concept [4]. In later research with parents of children with chronic, complex, and critical illness, the term “trying to be a good parent” has been further defined and described as a deeply personal, duty-directing term [5,6,7,8,9]. Parents have taught us that the personal “being a good parent” definition guides medical decisions and is influenced by medical encounters [8,9].

The “good-parent beliefs concept” has evolved and matured through the past two decades of learning with and from parent perspectives. In 2009, Hinds et al. established the good parent definition among 62 parents of children with cancer who engaged in one of three difficult decisions (enrollment in a phase I study, do not resuscitate status, or terminal care) in recent days [7]. In this landmark study, the good parent qualitative definition shared by parents contained the following themes: “making informed, unselfish decisions in the child’s best interest, remaining at the child’s side, showing the child that he is cherished, teaching the child to make good decisions, advocating for the child with the staff, and promoting the child’s health” [7]. In 2010, Maurer and colleagues identified that the elements of the good parent definition differ in frequency between parents of children with cancer who chose to enroll their child in a Phase I clinical trial and parents who instead chose a do not resuscitate decision [10]. The former group was more likely to cite medical facts and the need to prolong life, while the latter group was more likely to emphasize overall quality of life for the ill child and honoring the ill child’s wishes [10]. Both groups of parents emphasized doing what they believed was ‘right’ for their child in the current situation [10].

In 2012, Hinds and colleagues demonstrated the acceptability to parents of children with advanced cancer to complete a ‘good parent’ interview shortly after having participated in making an end-of-life treatment decision for their child [11]. All participating parents gave permission for their interview responses to be shared with their child’s health care team to help guide the team’s end-of-life care for their child [11]. In a subsequent follow-up contact to assess harm in participating in this interview, one parent reported finding it hard to relive the decision, approximately 28% reported being helped themselves by being in the study, and 56.6% reported being helped by believing their participation could help others.

In a study involving 78 parents of children with advanced cancer, eight themes related to communication emerged from the use of semi-structured interviews [12]. Six of the themes had been previously identified in research involving adult oncology patients and two were unique to the pediatric oncology study sample [12]. One of the unique themes was related to validating and reinforcing parental beliefs about having been ‘good parents’ to their very ill child [12]. The authors of this qualitative study urged for additional inquiry into this revealed ‘good parent’ theme [13].

In 2014, October et al. constructed the good parent definition beyond cancer diagnoses by studying the concept in the pediatric intensive care unit, noting “fathers ranked making informed medical decisions as most important, whereas mothers ranked focusing on the child’s health and putting their child’s needs above their own as most important” [8]. October’s work made special recognition of the ways sole maternal parents may prioritize good parent definitions as making informed medical decisions [8]. In 2015, Feudtner et al. used a novel ranking tool to study how 200 parents prioritize the good parent attributes [5]. This discrete choice experiment revealed groupings of similar patterns of good-parent–attribute ratings that were labeled as follows: “child feels loved, child’s health, advocacy and informed, and spiritual well-being” [5]. In 2019, Hill and colleagues expanded the “good parent concept” into a longitudinal exploration with recognition that some parents may change their parenting priorities over time, seemingly from an emphasis on knowledge to an emphasis on love [6]. In more recent years, Robinson et al. focused on father’s “good-parent beliefs” and strategically explored how staff may support fathers in the good-parent beliefs concept for paternal perspective and inclusivity [9,14].

Within these studies, parents have voiced appreciating opportunity to speak about their unique “good parent” philosophy as a source of support and guidance [5,7,8]. While research teams and review boards have worried that the concept of “being a good parent” risks the counter-label of “bad parent” [4], parent participants have repeatedly shared that the concept of “being a good parent” exists and evolves in real ways and have claimed the term as a real parenting phenomenon [4]. Far from the idea of perfect parenting or judgment toward parenting choices, the “good parent concept” is voiced by parents as being a term of affirmation and encouragement [2]. The “good parent” concept is one which fosters parents to recognize how this guides their parenting relationships, choices, and presence [15,16] with an intention of supporting parents.

Current gaps exist in the growing good parent concept research base, particularly in regard to exploring if, when, and how a parent becomes aware of his or her own definition of “being a good parent”; whether a parent perceives that this definition changes over time; what or who may influence a parent’s personal “good parent” definition; the ways “being a good parent” to an ill child may be the same or different than for well siblings; benefits or obstacles perceived by parents in striving to reach their definition; and whether parents perceive themselves as attaining their own unique “good parent” as they personally define this concept. Despite the growing evidence-based exploration of the “good parent concept”, these gaps represent communication voids in the current state of the science regarding this evolving concept [2]. This study explores these gaps in knowledge about the good-parent beliefs concept by learning more from parental perspectives.

## 2. Materials and Methods

### 2.1. Design

To address these questions, the survey protocol “Courageous Parents, Good Parents” was written. The study design was a one-timepoint online survey consisting of open-text and multiple-choice question formats. The target population for the online survey was parents of children (age birth to 18 years) with chronic or complex illness residing in the United States. Bereaved parents were included in the survey in parallel with parents of children living with life-impacting illness.

Survey questions were designed by a collaborative, interdisciplinary study team according to the Tailored Method of Survey Design [17]. Survey development included interviews with 10 bereaved parents and 10 parents of children living with chronic, complex, or critical illnesses to review questions developed by the study team with > 30 cumulative years of experience in pediatric palliative care. Feedback from the reviews by these 20 parents resulted in a revision of the survey questions to include branch logic so that bereaved parents encountered the same survey questions but in separate, parallel survey format from parents whose child was still living to reflect sensitivity in response options. Feedback from the pilot survey also resulted in the provision of a gentle introduction to the survey clearly stating that the term “good parent” did not equate to “perfect parent” and did not have a shadow, hidden “bad parent” meaning but instead inclusively honored the good intentions of all parents with celebration of diversity in parenting approaches. Parents recommended the incorporation of encouraging and positive parenting quotations embedded within the “advance survey” button to foster a less sterile survey experience and more aesthetic survey interface.

The survey was then independently reviewed, piloted, revised, and re-piloted by an interdisciplinary team (four expert parents from the Courageous Parents Network, two physicians, two social workers, two psychologists, two nurse scientists and one nurse case manager, one chaplain, and one mixed methodologist) prior to the survey launch.

### 2.2. Sample, Study, Site, and Population

This one-time, web-based survey study was distributed by the Courageous Parents Network (CPN) (https://courageousparentsnetwork.org/about/). The Courageous Parents Network (CPN) is a US-based nonprofit, 501©(3) organization, created by parents, for parents of seriously ill children, and by the providers who care for them, with oversight from professional advisory boards. The mission of the CPN is as follows: “To empower, support, and equip families and providers caring for children with serious illness.” Parents of children with chronic or critical conditions who are members of Courageous Parents were invited to participate in the online survey via email as well as through social media, with two reminder emails spaced 14 and 30 days apart. Due to the reach of the CPN website, participants represented parents of children with chronic or critical illness across the continental United States. A live virtual discussion hour featuring study team members was hosted by CPN in July 2020 to highlight the good parent concept (https://courageousparentsnetwork.org/pages/in-the-room-a-good-parent/) with a survey link shared. The survey was open for eleven weeks from June to August 2020.

### 2.3. Procedure

A secure RedCap© questionnaire format was utilized for online data collection. The final survey instrument consisted of a combination of 63 open- and closed-ended questions.

### 2.4. Analysis of Data

All quantitative analyses were computed using SAS software (SAS/ACCESS^®^ 9.4 Interface, Cary, North Carolina, 2013). The analyses were descriptive and univariate in nature. The study team applied counts for categorical variable responses. For missing responses due to skip patterns in the survey, the number of responders was used as the denominator (actual n). A total of 12 surveys were opened but not completed beyond the first three survey questions and thus were not included in data analyses. Frequencies and percentages are presented.

The question as to how often parents perceive they are able to be the “good parent” they want to be for their child consisted of the responses “*rarely*”, “*some days yes” and “some days no*”, “*often*”, “*always*”, and “*unsure*”. Due to zero responses in the *unsure* category, this variable was transformed into a three-level ordinal scale as follows: 0 = “rarely”, 1 = “often” and “some days yes and some days no”, and 2 = “always”. An ordinal logistics regression model was used to explore the relationship between the three-level ordinal variable and the child’s age and also the child’s length of illness.

Parent responses to open-ended questions were analyzed utilizing MAXQDA (VERBI Software, Berlin, Germany, 2020). A primary coder created a codebook and analyzed each phrase for content provided by the respondent thematically by question. A secondary coder reviewed these phrases for classification. Inter-rater reliability was notably > 85% between coders.

Binary variables were created for prominent themes related to respondents’ good parent definitions, and correlations were calculated for individual definition themes and a parent’s perception of how often they were able to reach their definition. Questions related to parents’ perceptions and feelings contained multiple response categories. These categories were reported as dichotomous variables using a Likert scale. Chi-square analyses were used to determine significant differences for each question between parents with a living child and bereaved parents. A probability of *p* < 0.05 was considered significant.

### 2.5. Ethical Considerations

The University of Nebraska (Omaha, NE, USA) Institutional Review Board (IRB) deemed the survey protocol as exempt from full IRB review (Study #197-20-EX). All participants gave their informed consent for inclusion by signing an electronic consent document before they participated in the study. The study was conducted in accordance with the Declaration of Helsinki.

## 3. Results

Survey response rate is unknown due to the inability to quantify parent-specific CPN social media followers or the potential sharing of the survey link by CPN members with other parents or with disease groups or others. Survey respondents included 60 biological mothers, five fathers, and two foster parents or legal guardians of sons (52%) and daughters (48%). The age of living children ranged from 4 months to 16 years with an average age of 7.6 years (duration of illness ranged from 3 months to 15 years with an average 6.7 years). Eleven bereaved parents shared that their children lived a mean lifespan duration of 6 cherished years. There was not a significant difference in the response patterns of bereaved parents and parents of living children according to quantitative analyses.

Characteristics of the sample include parents of children with a complex chronic medical condition who are members of the CPN. Participants were primarily Caucasian, college-educated mothers (90%) in a married partnership and co-parent role from suburban (58%) or urban (25%) geographies across the United States. Parent age was not asked. Respondent demographics are provided in Table 1.

### 3.1. Parental Responses Regarding Their Children’s Medical Needs

Pediatric diagnoses included metabolic and genetic (41%), neurologic (22%), oncologic (15%), pulmonary (15%), and gastrointestinal (7%) conditions. Two respondents shared that their children reside in an inpatient care setting with the remainder of children home with family. Home-based nursing hours were notably under-serviced with an average of 27 hours of home nursing hours approved per week but only an average of 16 nursing hours staffed. Two-thirds of parents depicted six or more medical subspecialists’ active involvement in their child’s care. Children spent an average of 21.5 days in an inpatient hospital setting over the past year. Thirty-nine percent of families had current palliative care involvement.

### 3.2. Defining the “Being a Good Parent” Concept

When asked, “How would you define ‘being a good parent’ to your child (child with medical diagnoses)?”, survey respondents provided open-ended responses that included their own personal definitions (Table 2).

### 3.3. Feelings about the “Being a Good Parent” Concept

When asked whether or not the term “being a good parent” resonated with them, 85% of participants answered in the affirmative. When asked in multi-choice format to describe feelings about the experience of being asked to discuss what it means “to be a good parent” to their child with medical illness, over half of the parents felt supported (55%) while some felt stressed (10%), relieved (5%), confused (2.5%), or were unsure about their associated feeling (27.5%).

### 3.4. Communication about the “Good Parent” Concept with Others

Of our respondents, 60% affirmed discussing their feelings about what it means ‘to be a good parent’ with their co-parent, other family members, friends, and other families of children with medical needs. In open-text response, parents mentioned that they discuss doubts, concerns, and daily struggles with others. Parents also shared that they reflect on their own ‘good parent’ definition with others to gain perspective and find ways to improve:

*“I discuss aspects of being a good parent with my mom, sister and close friends in order to gain perspective on choices that I make in parenting and life.*”

*“We talked as a couple as to what was the best possible thing we could do at the time, for our son. Which meant being the best we could be.*”

### 3.5. When the “Being a Good Parent” Concept Came into Parental Awareness

For the majority of parents, the concept of ‘being a good parent’ started to exist in parental awareness before the child’s birth (70.2%) or at the time of the child’s medical diagnosis (21.3%), although a few parents also reported the concept coming into existence during the child’s time of wellness (2.1%), at a key timepoint in the child’s illness (2.1%), or other timeframes (4.3%).

### 3.6. Ways the “Being a Good Parent” Concept Informs Interactions with the Child 

Content analyses of how the “good parent” concept informs the parent’s interactions with their medically complex child revealed the following themes: providing and prioritizing love, fostering positivity, offering physical affection and consistent presence, advocating for the child to be his or her best self, providing care and personalized communication, being both planful regarding the child’s needs and patient with the child, and cherishing time together.

**Figure 1:**
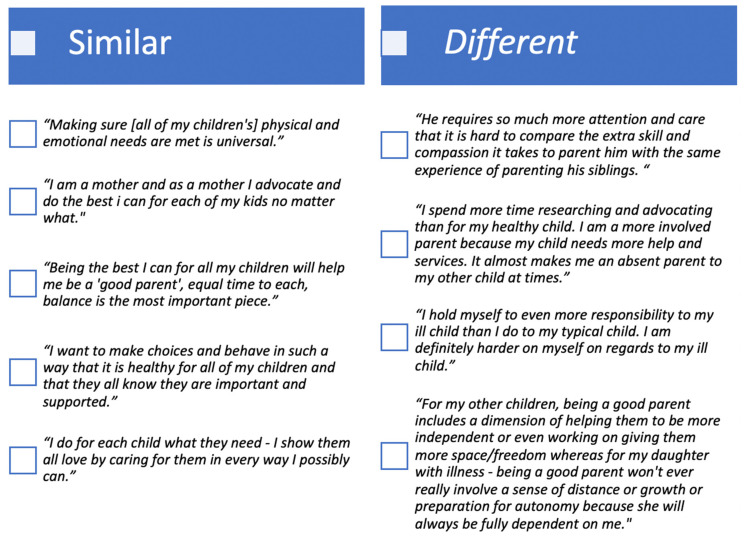
Differences and Similarities in ‘Good Parent’ Definition for Ill Child and Well Siblings.

For the 68% responding parents with more than one child, 46% responded to a binary yes/no question that their definition of ‘being a good parent’ to their ill child was the same as for the child’s siblings, whereas 54% perceived their definition of “being a good parent” as different for the ill child. In open-ended responses, parents described the ways that their “being a good parent” definition was the same or unique for the ill child with a medical condition and the child’s well siblings (Figure 1).

### 3.7. Factors of Influence on the “Good Parent” Concept

According to multiple-choice responses, items that influenced the personal “being a good parent” definition included the following: awareness of the child’s prognosis (43.5%); changes in the child’s health (37.7%); family interactions (31.8%); spiritual considerations (24.7%); clinician behaviors (18.8%); physician interactions (14.1%); nurse interactions (11.8%); and other influencers inclusive of the child’s preferences and lessons from other families of children with medical needs (15.3%).

### 3.8. Benefits and Challenges to the “Good Parent” Concept

**Figure 2:**
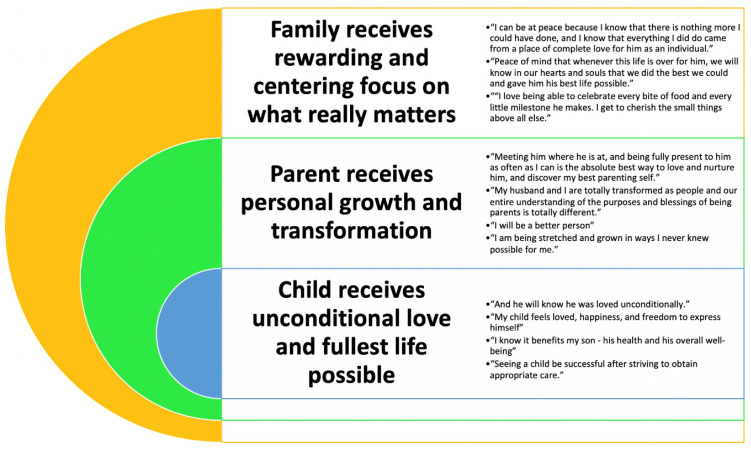
Benefits of personal “good parent” definition.

Parents were asked in open-text response to describe the challenges and benefits of the striving “to be a good parent” concept. Challenges included the following: the tangible provision of complex care in terms of time and managing hands-on care needs; the navigation of the multiple needs and opinions of all family members; the diverse perspectives and opinions of medical professionals; the inability for others to fully understand the family’s situation and subsequent sense of loneliness, judgement, or exclusion; internal expectations and self-judgment; progression of the child’s illness or symptom burden and concern for increase in the child’s suffering; and structural obstacles including difficulty accessing necessary services, resources, medications, case management, and support. Despite these very real and heavy challenges, profound benefits were reported as being experienced by many parents as part of working toward fulfilling the desire ‘to be a good parent’ to the child (Figure 2).

### 3.9. Changes in the Personal “Good Parent” Definition

When asked in a binary (yes/no) question about whether their personal definition has remained the same or changed over time, the majority of respondent parents reported that their definition of ‘being a good parent to my ill child’ changed over time (67.5%) versus 32.5% who felt that their definition remained the same longitudinally. For those depicting change in open-text response, this predominantly entails a transition to focusing more on quality experiences and memory-making as time passes. Parents describe their definition of ‘being a good parent’ shifting over time from having perfectionist ideals and stringent expectations of oneself to recognizing what is most important, namely “play, laughter, love, and comfort.” Exemplary quotes in this regard include the following:

*“I started parenthood with high ideals, and as I have experience[d] the serious illness of both my son and husband I have learned to recognize what is truly important—love, compassion and connection.*”

*“Because there is so much we cannot do for our son, we have had to adapt to realistic and attainable expectations for ourselves in order to feel empowered and successful in making what difference we can.*”

*“When she [our daughter] was very little, I thought that being a good parent for her meant being 100% committed to knowing absolutely everything about her medical condition—reading journal articles late at night, etc. Over time I realized that I couldn’t sustain that kind of focus and continue to build good relationships with her, her siblings, my spouse, or pretty much anyone else, so I began focusing more on sharing experiences with her.*”

*“Because of our son’s level of disability, we are not required to raise a child to be a productive member of society in the same way other parents do. There is less focus on right and wrong and more focus on love and comfort.*”

Being less worried about the judgment of others and also becoming less judgmental of others were depicted as an additional change in their “good parent” definition. Parents described giving themselves grace, seeking and receiving help, and recognizing the role for self-compassion and attending to their needs as an evolution in their ‘good parent’ definition:

*“It [my definition of “being a good parent”] has changed because I noticed I can’t do it on my own. I need help.*”

*“I have also had to be easier on myself and ease up on the expectations I have for myself.*”

*“Self-care and self-compassion became part of my definition.*”

*“I’ve learned to give myself grace. [My daughter’s] happiness is enough sometimes.*”

### 3.10. Attaining the Personal “Good Parent” Definition

Parents indicated feeling they were able to “be the ‘good parent’ they want to be for their child” “some days yes and some days no” (51.3%), often (25.6%), always (18%), or rarely (5.1%). Respondents who felt they were able to reach their good parent definition on some days often explicitly recognized that they sometimes experienced sadness, exhaustion, and a sense of feeling overwhelmed, but that they just tried again the next day. Other parents’ perception of whether they were meeting their ‘good parent’ definition was notably linked to how the child was doing on a particular day: *“varies by how she is doing*”; *“sometimes if his symptoms are bad that day, it’s hard to feel like I was the good parent because I couldn’t fix it for him and that matters to me and to him*”; *“it depends on my child’s mood and health.*” Parents who reached their definition “often” or “always” generally recognized that they were not perfect but that they strove to show the child every day what they thought was important and focused on enjoying the child:

*“We all have good days and bad days, but in the whole we have a happy child who is secure in our love. Health and comfort can be nebulous, but when times are hard, he doesn’t face those challenges alone.*”

*“It’s about the mindset of maintaining his best interests, not the steps. it’s not making soup or playing games or even being his confidante. It’s about his trust that I am on his side first and always.*”

*“I feel like as long as we have his best interests at heart we will always be good parents.*”

*“God designed her beautifully. She seems to have accepted the challenge (heart defect) and have high pain tolerance (even now, teething, no crying or whining). She’s smart and everything she does, and even her every facial expressions bring so much joyfulness to my heart. Often, I was not ready to say good night to her.*”

The defining concepts within the personal ‘good parent’ definition impacted whether a parent perceived they had reached their ‘good parent’ definition. In particular, the individual theme of *advocacy* had a correlation of 0.20 with parental sense of reaching their definition of ‘being a good parent’ often or always.

Based on the logistic regression analysis, an increase in the child’s age was associated with an increase in the odds of feeling a sense of “good parent” definition achievement with odds ratio of 1.3 (95% CI, 1.03 to 1.66, *p* = 0.03). Similarly, a longer duration of the child’s illness was associated with an increase in the odds of more often feeling a sense of being a good parent, with odds ratio of 1.27 (95% CI, 1.02 to 1.57, *p* = 0.035). Longevity (age) and chronicity (longer duration of illness) provided time to allow the parent to perceive they had reached their “good parent” definition.

## 4. Discussion

The results from this study suggest parental participants of children with medical complexity do maintain an internal working definition of what it means to be a “good parent” to their child. The majority of parent respondents believe that their “good parent” concept came into existence before the birth of their child and evolved over time. The defining concepts within the personal “good parent” definition collectively included the following: advocacy, love, care, comfort, creating meaningful memories, seeking fullest potential, striving for balance, gaining medical insight, and respecting the child. These themes resonate with prior research of the “good parent concept” among parents of children with terminal oncology diagnoses [7,9] and critical illness [8]. While almost half of respondents emphasized similarities between their “good parent” definition for their child with complex medical needs and their other children, parents recognized that the needs of their ill child were uniquely different and parenting the child with medical needs required special parental presence, knowledge, skill, communication, and advocacy.

### 4.1. A Novel Theme

A previously unreported theme of “finding a balance” was mentioned by parents in this study. Our respondents noted the importance—and difficulty—of striking a balance between keeping their child safe and letting them live as “normally” as possible; of meeting their child’s medical needs, but also treating them as “a child first”; of being there for their medically complex child but also for their other children, their partner, and/or their work. For some parents, striking this balance is an important part of “being a good parent”, a novel finding for the existing “good parent concept” research base.

### 4.2. An Early and Evolving Concept

For most of our respondents, a “good parent” definition came into awareness early in the child’s life and evolved over time based primarily on the child’s changing needs; familial, environmental, and medical care influences; and parental growth. The survey did not inquire into whether parents knew of the child’s medical condition in the fetal period, as this would have been an interesting correlation with when parents became aware of their unique “good parent” definition [18,19].

### 4.3. Attaining the Concept

The finding that parents of older children or children with longer duration of illness were more likely to perceive they had attained or reached their internal definition of “being a good parent” hints at the longitudinal nature and progression of the “good parent” concept in actual daily life. Increasing familiarity with the child’s unique needs and preferences or likes over time may foster parental sense of “good parent” internal attainment. Or, as has been explored in a prior longitudinal study, parental shifting from focus on medical knowledge at time of diagnosis to prioritization of loving the child may be part of this perceived progression toward goal attainment [6].

This study’s finding that parents of older children and parents of children who had lived with their diagnoses for longer duration were more likely to perceive having reached or attained their “good parent” definition warrants further exploration of the experiences and perceptions of parents of young children, particularly children who succumb to illness in the neonatal period and parents of children who experience sudden diagnoses with shortened lifespan.

The idea of “reaching” or “attaining” the personal “good parent” definition included growing into a personal goal but also included the gentle process of acceptance that “perfect parenting” or the parenting expectations of others is not the same as an authentic “being a good parent” internal goal. Further work is warranted to consider ways in which parents may receive affirmation or encouragement in their “trying to be a good parent” goals. Parents in this study reflected on doing their best day by day, while bringing the courage and compassion needed to continue trying again each following day on behalf of their loved child.

### 4.4. Advocacy as Central

The trend between the theme of *advocacy* and parental perception of having reached their internal “good parent” definition may be credited in part to a sense of daily reinforcement as parents witness their child achieving successes, meeting milestones, accessing medical services or therapies, or obtaining support resources as a result of parent advocacy. Hence, advocacy is something that a parent may not actively have to “do” every day, but it is something the results of which they may be witnessing frequently. Future research would necessarily include exploration into how parents define advocacy for their individual child and within their family unit. The idea of medical team members partnering with parents in advocating for a child’s care needs is recognized as a core value in esteemed therapeutic relationships [20,21].

### 4.5. A Role for Palliative Care

The medical complexity of the children represented in this study was notably high with the involvement of supportive interventions and technologies and the inclusion of many medical subspecialists due to care needs. Lack of filled home nursing hours (40% allocated nursing support hours unfilled) translates into many respondent parents carrying both relational and direct hands-on medical roles for their child with medical needs. Parents were notably navigating a biomedical and relational role for their child. Pediatric palliative care teams were under-represented in the children’s care circles with over one-third of families never having had palliative care involvement. This represents a missed opportunity for support for these children and families [22], particularly as over one-quarter of respondent parents “were unsure” about their feelings surrounding their “good parent” goals. The field of pediatric palliative care is committed to working with families to explore the guiding family values, relational needs, medical support, and goals of care which matter most to families [23,24].

### 4.6. Limitations of the Study

Limitations of this study include a one-timepoint format without longitudinal tracking. The survey was not a validated instrument. The survey was designed to explore the “gaps” in the current knowledge base of the “good parent concept” and iteratively developed and piloted through a participatory research approach with parents and interdisciplinary stakeholders. The inability to quantify response rate is a recognized study limitation as was the exclusion of incomplete surveys. Respondents were primarily Caucasian co-parenting females from Christian tradition with post-secondary education. The good parent concept is notably influenced by socioeconomic status, gender, familial, cultural, and social determinants of health, warranting further focus on theme exploration in diverse communities [4,8]. The nature of survey research risks response and selection bias, although the findings from this survey were congruent with prior “good parent” thematic exploration [4].

## 5. Conclusions

This study suggests that the concept of “being a good parent” does exist early and evolves over time for parents of children with complex medical needs, serving as a guide for the way the parent interacts with the child and approaches the child’s medical care and even experiences bereavement. Parents do reflect on how often they are “attaining” their definition of “being a good parent” in a way that warrants careful consideration for medical teams in providing support, co-advocacy, and high regard for parents’ courageous, caring, and good intentions toward their loved children.

## Figures and Tables

**Table 1:** Demographics table.

Category	Response (*n* = 67)	%
Relationship to Child		
	Mother	(89.55)
	Father	(7.46)
	Foster Parent or Guardian	(2.98)
Parenting Role		
	Lone Parent	(12.5)
	Co-Parent	(87.5)
Marital Status		
	Married	(87.5)
	Divorced	(7.5)
	Single	(5)
Number of Children		
	One	(32)
	More than one	(68)
Parent Race/Ethnicity		
	Asian	(5)
	Black/African	(2.5)
	Caucasian	(90)
	Other	(2.5)
Parent Religion		
	Christian	(59)
	Jewish	(5)
	Agnostic/Atheist	(13)
	Spiritual but not religious	(15)
	Other	(8)
Family Geography		
	Urban	(25)
	Rural	(16.67)
	Suburban	(58.33)
Parent’s Highest Education Level		
	Some College	(20)
	Completed College	(37.5)
	Graduate School	(42.5)
Insurance Coverage for Child		
	Medicaid	(20.51)
	Private Insurance	(30.77)
	Both	(48.72)
Parent Employment		
	Part-time	(12.5)
	Full-time	(42.5)
	Not employed outside of the home	(25)
	Home-based employment	(20)
Family Financial Concerns		
	None	(40)
	Some	(37.5)
	Many	(15)
	Prefer not to answer	(7.5)
Child Status		
	Alive	(83.82)
	Deceased	(16.18)
Child Gender		
	Male	(52.08)
	Female	(47.92)
Child Medical Support		
	Tracheostomy *	(14.9)
	Gastrostomy Tube *	(81.3)
	Central Line *	(52.2)

* Designates current or prior technology.

**Table 2:** Good Parent Definition Themes and Quotes (*n* = 45 parents with free-text responses).

Theme	Respondents	Exemplary Quotes
Being an advocate Championing, supporting, and backing the child; obtaining access to therapies and equipment on behalf of the child	16 parents	“It meant being an advocate for her in and out of the hospital” “Advocating for them, being their voice when they can’t” “Understand doctors are not Gods and are often wrong with chronically ill kiddos. Fight for your child’s needs and what you believe will help that child obtain better care in home, hospital, and school settings”
Conveying love to my child Unconditional endearment and devotion; the child always knowing that they were cherished	12 parents	“My goal was that he also knew he was loved” “I try to focus on snuggles, holding her close, talking to her so she knows she’s always loved and safe” “I try to always love first. Make decisions with love.”
Providing best possible care for my child Managing medications, appointments, therapies, etc.; being planful regarding complicated needs; attentiveness for the child’s well-being	11 parents	“Keeping up with all therapies and medical needs” “Stay on top of her meds & supplies, doctor appointments” “What I picture as being a good parent (playing, reading, singing, snuggling, teaching what is right and wrong) added to what I also have to do to keep him here (extensive treatments, medications, etc.) compete with each other in my head, I have to do work to merge and accept that these two things aren’t mutually exclusive.”
Comforting my child Offering physical closeness and snuggles but also controlling pain and alleviating symptoms and soothing the child	9 parents	“Focusing on what makes him comfortable” “If I can try to alleviate his symptoms during his decline, and if my efforts to minimize pain and suffering make a difference, I’m going to let that count.” “The biggest thing that I can do for his comfort is trying to connect with and include him, even in a vegetative state—which means baths, massages, books, snuggling, walks, talking and singing to him, and making sure he knows I am present.”
Creating meaningful memories with my child Engaging in rich and fun memories; spending special time with the child; pursuing positive moments while cherishing time	9 parents	“It may be creating extra fun memories that are special just for that child.” “Memories to share, even if those are sometimes involving medical needs” “I feel like a good parent when I make sure we have silly family jokes and fun memories to share.”
Helping my child reach fullest potential Fostering the ability for the child to be his or her best self	8 parents	“Giving her the tools and opportunities to take her as far as she can go in this life” “Make him as successful as he could be with his many limitations he was born with” “Allowing her to be her best self”
Seeking some balance amongst my many roles Incorporating multiple dimensions while avoiding extremes; coordinating medical with emotional needs; incorporating needs of the ill child with the needs of their siblings; equilibrating safety and letting the child live a normal life	8 parents	“Balancing his education and care needs with the needs of my other children, myself and my spouse so that we are all able to be our best selves” “Balancing my hospital hours with at home hours” “Being his nurse, mom, play mate, and juggling many roles.”
Developing my own medical insights Learning about the child’s condition; studying and seeking information; advocating for the best possible medical care	8 parents	“Studying their diagnoses” “Doing my own research to help her” “Understanding their condition, educating myself on the latest research and challenging her specialists when necessary”
Respecting my child as a person Honoring a child’s independence and wishes; treating the child as a dignified child first; and being open, patient, and truthful with the child	7 parents	“It meant that while we were conscious of keeping information age-appropriate, it also meant that we would never lie to her” “Acknowledging and addressing him like a valid person” “We get her insight on things before making decisions together”
Weighing my child’s possible quality of life Considering quality of life issues and the implications of procedures and medications	7 parents	“Medical procedures that improve their quality of life” “Best quality of life possible” “Think about long-term effects of procedures or medications”
